# Constructive neutral evolution: exploring evolutionary theory’s curious disconnect

**DOI:** 10.1186/1745-6150-7-35

**Published:** 2012-10-13

**Authors:** Arlin Stoltzfus

**Affiliations:** 1 Institute for Bioscience and Biotechnology Research, 9600 Gudelsky Drive, Rockville, MD 20850, USA; 2 Biochemical Science Division, NIST, 100 Bureau Drive, Gaithersburg, MD 20899, USA

**Keywords:** Evolutionary theory, Constructive neutral evolution, Neo-Darwinism, Mutation, Evolutionary genetics, Mutation bias, Modern Synthesis

## Abstract

**Abstract:**

Constructive neutral evolution (CNE) suggests that neutral evolution may follow a stepwise path to extravagance. Whether or not CNE is common, the mere possibility raises provocative questions about causation: in classical neo-Darwinian thinking, selection is the sole source of creativity and direction, the only force that can cause trends or build complex features. However, much of contemporary evolutionary genetics departs from the conception of evolution underlying neo-Darwinism, resulting in a widening gap between what formal models allow, and what the prevailing view of the causes of evolution suggests. In particular, a mutationist conception of evolution as a 2-step origin-fixation process has been a source of theoretical innovation for 40 years, appearing not only in the Neutral Theory, but also in recent breakthroughs in modeling adaptation (the “mutational landscape” model), and in practical software for sequence analysis. In this conception, mutation is not a source of raw materials, but an agent that introduces novelty, while selection is not an agent that shapes features, but a stochastic sieve. This view, which now lays claim to important theoretical, experimental, and practical results, demands our attention. CNE provides a way to explore its most significant implications about the role of variation in evolution.

**Reviewers:**

Alex Kondrashov, Eugene Koonin and Johann Peter Gogarten reviewed this article.

## A curious disconnect

**Figure 1:**
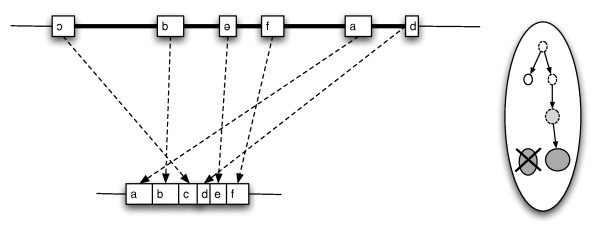
**Gene scrambling. **In a post-mating ciliate cell (right), the micronucleus divides, and one daughter is converted into a new macronucleus (gray circle) as the old macronucleus (gray oval) degenerates. Processing to generate macronuclear genes (left) includes removal of intervening segments (black bars) and unscrambling of segmented genes.

Occasionally, nature startles us with baroque and apparently gratuitous complexity. Several recent articles [1-5] have drawn attention to “Constructive Neutral Evolution” (CNE), a scheme proposed to account for such conspicuously nugatory features as gene-scrambling (Figure 1) and RNA pan-editing, for an inordinately complex spliceosome, and for a profusion of semi-redundant duplicate genes [6,7].

It is hard to imagine an idea less congenial to the adaptationist habit of thought that has dominated biology from pre-evolutionary times. The classic paradigm of an evolutionary explanation is an adaptive rationale: feature X exists by virtue of benefit Y. If one version of “Y explains X” doesn’t work, the adaptationist imperative compels us to revise the rationale (Y’), or re-define the feature (X’), a cycle that may be repeated indefinitely [8].

Skeptics— from Bateson a century ago [9] to Lynch [10] today— have questioned the value of adaptationism as a research strategy, but it was not until Gould and Lewontin [8] famously deconstructed the “adaptationist research program” that this skepticism began to coalesce into an alternative view. In the reformist counter-paradigm, one invokes “chance”, “constraints”, and “history” to explain imperfections: some features don’t turn out perfectly, due to statistical noise, in-built limitations, and so on; some features, due to “historical contingency”, are side-effects or vestiges. Selection still governs evolution, as Darwin said, but there are “limits to selection” [11].

CNE seems to provide something completely different: not features that are noisy, limited manifestations of an adaptive reason-for-being, but complex features that emerge by an internal dynamic without an adaptive *raison d’être*. Our neo-Darwinian intellectual ancestors— who derided the notion of internal forces as an appeal to vitalism— would have scoffed at CNE.

Yet if we peek inside CNE, there is no magic. The most well known model of this type is the neutral sub-functionalization model for the promulgation of duplicate genes proposed independently by Stoltzfus [7] and by Force, Lynch, et al. [12,13], who refer to it as the duplication-degeneration-complementation (DDC) model. In the conventional view, opportunities for adaptive specialization drive the expansion of gene families; in the DDC model, duplication creates a capacity for complementation of mutations that impair sub-functions, allowing duplicate pairs to stumble from redundancy to co-dependency, resulting in apparently specialized genes. DDC seems to have sparked a minor renaissance of interest in gene duplication models— Force, et al. have been cited over 2000 times! Though controversial, there is no doubt that the model— which has been formalized and evaluated by computer simulations and mathematical modeling— is a legitimate implication of evolutionary genetics [14].

How could evolutionary genetics produce something so odd that it fails to fit, not only the classic adaptationist paradigm, but also the reformed constraints-chance-contingency paradigm?

The answer relates to what Orr [15] calls the “curious disconnect” between the “verbal theory that sits at the heart of neo-Darwinism” and the emerging theoretical implications of evolutionary genetics. The roots of this disconnect may be found in a long historic struggle to reconcile evolutionary thinking with the emerging facts of heredity [16]. After the discovery of Mendelian genetics in 1900, this struggle took the form of a dispute between 3 concepts, all confusingly called “selection”: (1) Darwin’s pre-genetic concept of a creative force that molds traits from a blending mass of infinitesimal differences (raw materials); (2) a Mendelian frequency-shifting force that acts on true-breeding types, so that a rare type may replace a common one; and (3) a stochastic filter (a sieve or pruning hook) that modulates the chance of survival of spontaneously arising mutants, some of which get lucky.

The battle lines of this historic conflict continue to shape contemporary evolutionary discourse. Darwin’s original theory was based on a non-Mendelian view of heredity characterized by blending of environment-induced, continuous variation [16,17]. When confronted with the complaint that selection is not creative, but merely addresses “the relative success and failure of such new forms as may be born into the world”, Darwin’s response was clear: “that may be a very good theory, but it is not mine” [18]. That is, while we may think of concepts #1 to #3 above as manifestations of an elemental Darwinian principle of selection, Darwin and his followers did not agree, and saw these as separate theories of evolution. This is why Darwin’s early followers resisted Mendelism, and rejected the mutationist view (#3) of de Vries, Bateson, and Morgan as an anti-Darwinian view without selection (i.e., without #1).

The Modern Synthesis (MS), or modern neo-Darwinism, emerged from the claim that Darwin’s shape-shifting force (#1) could be reconciled with the Mendelian frequency-shifting force (#2) by arguing that a Darwinian process (smoothly shifting a mass of blending differences) occurs at the phenotypic level because, at the genotypic level, Mendelian selection is shifting the frequencies of discretely inherited (non-blending) allelic types at many loci, each with an infinitesimal additive effect on the phenotype [16,19]. This reconciliation, often credited to Fisher and referred to as the Fisherian view, led to the branch of theory known as quantitative genetics, dealing with quantitative traits, e.g., the height of a plant or the fat content of its seeds. On this basis, Fisher, Mayr and the other architects of the MS resurrected Darwin’s verbal theory, replacing Darwin’s non-Mendelian view of heredity with the new idea that each species has a “gene pool” that maintains abundant, uniform, infinitesimal variation to serve as raw materials for selection.

That was a risky position, because the possible domain of Mendelian evolutionary genetics is (in principle) much broader than that under which the essential claim of the MS— that Mendelism rationalizes “natural selection” (#1)— is valid, as implied in a review of quantitative genetics [20] that warns:

"If stochastic events, such as genetic drift, fluctuating adaptive landscapes and rare mutations, are more important, then quantitative genetics might not be informative and macroevolution might be decoupled from microevolution. Resolution of this issue is crucial to evolutionary biology as a whole (p. 322)."

In Darwin’s original theory, and in the later Fisherian view, individual differences are properly a raw material, like the sand used to make a sand-castle: each individual grain of sand may be unique in size and shape, but its individual nature hardly matters, because it is infinitesimal in relation to the whole that is built by selection. By contrast, if an episode of evolution reflects the individual nature of a significant mutation— a developmental macromutation, a gene or genome duplication, an event of lateral transfer or endosymbiogenesis, etc.—, then the infinitesimal assumption no longer applies, and the verbal theory fails: when variation supplies form (not just substance), it is no longer properly a raw material, and selection is no longer the creator that shapes raw materials into products.

That is, modern neo-Darwinism (the MS) is a Mendelian rationalization of Darwin’s “natural selection” (#1), but the conditions of this rationalization are narrow, and “the verbal theory that sits at the heart of neo-Darwinism” begins to break down when these conditions are unsatisfied.

The concepts of “chance”, “contingency” and “constraints” allow the original theory to be stretched to fit cases outside of its narrow proper domain. Yet, these flexible concepts don’t change the underlying paradigm— neo-Darwinism plus excuses is still neo-Darwinism—, and this sometimes results in logical gaps. For instance, to suggest that the lack of variation is a constraint or limit on selection only makes sense in regard to Darwinian “natural selection” (#1); while in regard to the other concepts (#2 or #3), invoking the absence of a variation as a constraint or limit is nonsensical, like saying that the absence of mass is a constraint on the force of gravity.

That is, sometimes the gap becomes a disconnect, which brings us back to the “curious disconnect” invoked by Orr in regard to the “mutational landscape” model of adaptation. In this model of evolutionary change by discrete steps, movement in a potentially huge genotypic space is reduced to repeatedly applying a purely local move-rule to the sub-space within 1 mutational step of the current genotype. Orr is trying to explain why this model— which seems obvious in retrospect— was not developed until the 1980’s, and then was ignored for over a decade. He argues that the belief that the problem of adaptation was solved by Fisher’s infinitesimal model stifled further research by defining away all the interesting questions: "we cannot, after all, construct a meaningful theory of adaptation if we assume away the existence of mutations that have different-sized phenotypic effects" [21]. This recalls the sarcastic critique made a century earlier by Bateson, one of history’s foremost mutationists, whom Orr quotes:

By suggesting that the steps through which an adaptive mechanism arose were indefinite and insensible, all further trouble is spared. While it could be said that species arise by an insensible and imperceptible process of variation, there was clearly no use in tiring ourselves by trying to perceive that process [9].

Indeed, the mutational landscape model treats selection as a stochastic sieve, reflecting a conception of evolution more in line with the thinking of de Vries, Bateson and Morgan, than of Darwin, Fisher and Mayr (Orr literally invokes “lucky mutations” [22], the concept ridiculed by the architects of the MS, e.g., p. 101 of [23]). The same may be said for the Neutral Theory, CNE, and some other post-MS innovations mentioned below: they all represent evolution in terms of events that follow origin-fixation dynamics, reflecting a conception of evolution as a 2-step proposal-acceptance process.

Such models clearly emerged from within a scientific culture dominated by neo-Darwinism and the MS, and yet, conceptually and historically, they are not part of the MS: they emerged well after the MS was established, they fail the test of concordance with Darwin’s stated views— which is why Darwin’s followers correctly judged mutationism as an anti-Darwinian view—, and their behavior is not well described by “the verbal theory that sits at the heart of neo-Darwinism”. If we are to understand the behavior of these models, we must imagine a different verbal theory describing the causal roles of selection and variation.

## Scrambling cords and genes

One of the curiosities addressed by an original CNE model [7] is gene scrambling (Figure 1). The model, which depends on idiosyncratic Ciliate-specific features of the gene organization, is easier to understand if we begin with a more familiar system— desk organization.

**Figure 2:**
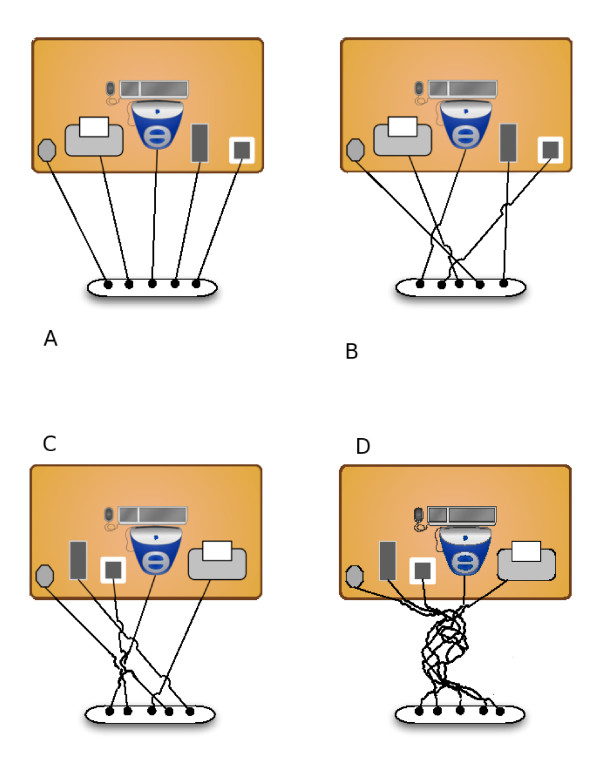
**Cord scrambling. **Arcs show where one power cord passes over another, thus in (**B**), the cords with no arcs are below all others, while those in power outlets 1 and 2 are above all others. The devices in (**B**) can be removed in the order (1 or 2) before (3 or 5) before 4, which must be removed last (numbering the devices in left-to-right order of power outlets). In (**C**), device 5 may be removed first, then device 3, but the remaining devices are tangled. In (**D**), all devices are tangled.

Imagine some computer devices (monitor, printer, router, etc.) arranged on a desk, with their power cords plugged into sequential outlets on a power strip. A fastidious person would arrange the devices first, and then install the cords in the same left-to-right order (Figure 2a). This is the minimally tangled configuration of cords; in formal terms, the cords are topologically unlinked. This means that, approaching the desk from behind, any device may be removed directly, without untangling, by grabbing the device with one hand, and the plug with the other, and pulling. A less fastidious person might simply install the devices one at a time, yielding a configuration with low (but not minimal) linkage, such that the devices may be removed without untangling, if they are removed in the reverse order of installation (Figure 2b).

Over time, however, there are many ways that linkage may increase to the point that some devices cannot be removed without untangling (Figure 2c). For instance, we may decide to swap the printer and the router, tangling their cords together in the process. Over time, tangling will increase to the point that no device can be removed without untangling. Ultimately, without deliberate remedial action, we get the familiar experience of power-cord dreadlocks (Figure 2d).

Note that we cannot account for cord-tangling by invoking local benefits. Imagine that, in order to make more space for the mouse, we move the printer to the other side of the monitor, tangling the cords in the process. Over time, we may make many such improvements. However, while optimization of our desktop organization via device movements would *entail* the tangling of cords, *optimization per se* is not the cause of tangling, because the same tangling would happen even if we repositioned devices arbitrarily, with no objective. Indeed, so long as our reasons for moving devices are unrelated to the topology of the cords— if we move devices on top of the desk, without looking below it—, it is as if these movements were “neutral”.

Instead, we can explain the tangling of cords by noting that, for any given order of devices, there is only one fully unlinked topology of cords, and an enormous universe of increasingly tangled topologies. The global or ultimate cause of tangling is that the system starts out in a topologically distinctive untangled configuration; the corresponding local cause for initial increases in tangling is that the set of available device movements (e.g., pass device 1 under device 3) provides more ways to increase tangling rather than to decrease it.

With the example of cord-tangling in mind, let us consider the bizarre case of gene-scrambling in Ciliates, single-celled organisms with both a small germ-line micronucleus, and a larger somatic macronucleus, in which gene expression occurs. Thus, information transfer in ciliates goes, not DNA→RNA→protein, but micDNA→macDNA→RNA→protein. The extra step provides a niche (in genome ecology) for selfish elements with a propensity to excise during macronuclear development (i.e., like introns, except spliced out from DNA). Ciliates have such elements in abundance: germ-line genes are split into macronuclear-destined sequences (MDSs) by internal eliminated sequences (IESs) that are excised in development (Figure 1).

**Figure 3:**
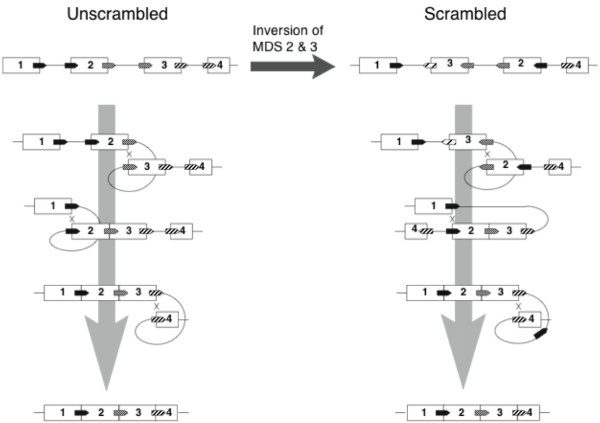
**IES removal via recombination of paired key (pointer) sequences provides a gratuitous capacity for unscrambling. **Here, an inversion-scrambled gene is processed with precisely the same 3 crossovers (“X” marks) as its unscrambled parent. Large downward arrows, developmental processing to generate macronuclear genes; rightward arrow, evolutionary change.

Furthermore, in some ciliates, the MDSs of a gene may be scrambled in order and orientation [24] (Figure 1). The CNE model for gene scrambling [7] is based on a side-effect of the postulated mechanism for removing IESs using flanking “key” or “pointer” sequences, similar to the repeats generated by various types of transposons. As illustrated in Figure 3, this mechanism confers a gratuitous capacity to unscramble genes scrambled by inversions or translocations. The pointer sequences automatically restore the correct ordering of fragments during developmental processing of IESs. To the extent that this forestalls the otherwise deleterious effects of rearrangements, mutations to scrambled configurations would be neutral mutations subject to fixation by drift.

In the language of CNE, the initial capacity for developmental unscrambling— presumed in an ancestor prior to the first scrambled gene— is called an “excess capacity”, meaning a capacity that could be removed (in principle) without compromising fitness.

Likewise, in the CNE gene duplication (DDC) model, the excess capacity is the capacity of one duplicate copy to compensate for defects in the other. In the CNE model for spliceosome complexification, an ancestral intron is presumed to have the gratuitous capacity to reassemble and splice when split into pieces— as shown experimentally for group II introns and even for protein-based inteins [25]— , and this allows it to evolve into the multiple snRNAs of the spliceosome. Biological systems of all sorts have excess capacities, e.g., a recent commentary marvels at the gratuitous capacities of metabolic networks [26].

While an excess capacity, by definition, is not “functional”, this can be changed. For instance, once a scrambled gene has evolved, the capacity for unscrambling is no longer a side effect but— by the normal standards of biological discourse— a “function”, conserved by negative selection against mutations that would compromise it.

**Figure 4:**
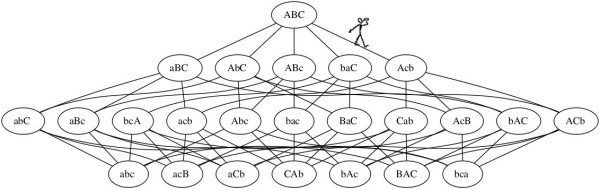
**A drunkard’s walk through scrambling space. **Ovals show possible configurations for a gene with just 3 MDS segments A, B and C. Inverted segments are shown in lower-case (e.g., inverting block AB converts ABC into baC). Lines between ovals represent evolutionary paths (mutation-fixation events) from one configuration to another. The figure shows only inversion paths; considering translocations would add some new pathways (e.g., a link from ABC to BAC), but no new configurations. This graph (minus the drunkard) was computed automatically by a Perl script (available as Additional file 1) and plotted with GraphViz.

Excess capacities and negative selection, though essential in CNE models, do not provide the directionality to account for a tendency toward increased scrambling. To understand this, we may note that for a ciliate gene, there is a single unscrambled configuration, and an enormous number of increasingly scrambled configurations (e.g., for n = 6 segments, there are 2^n-1^n! = 23040 configurations). Just as for the case of cord-tangling, scrambling is favored for entropic reasons. Over time, if a scrambled gene can evolve (by mutation and drift), it will evolve, and by subsequent changes it will be less likely to revert than to wander, by a drunkard’s walk, more deeply into a vast space of scrambled possibilities (Figure 4).

The potential for local adaptive benefits does not necessarily change this directionality. That is, as in the case of the cord-tangling, where we might optimize devices without looking under the desk, there may be biological optimization principles that do not “see” MDS order, and thus represent orthogonal factors. For instance, a mutation that inverts or translocates a segment of micronuclear DNA might result in more effective replication or chromosome segregation in the micronucleus (where genes are not expressed), and it may undergo selective fixation due to this effect. A small subset of these beneficial changes will invert or translocate an MDS segment, increasing or decreasing scrambling. Other things being equal, we expect such beneficial rearrangements to lead, over time, to extensive scrambling.

Finally, note that the tendency toward scrambling is just that— a tendency. Changes to a less scrambled state are possible. Furthermore, just as one might take remedial action to untangle the cords under a desk, nature is capable of dramatic reversals: an extensively scrambled ciliate gene could become unscrambled in one step by fixation of a mutation that introduces the unscrambled macronuclear DNA into the micronuclear genome.

## Rebels without a cause

The ultimate cause of scrambling, in the CNE model, is not an adaptive rationale, but a dynamic in which a gratuitous unscrambling capacity opens the door for the system to wander into a morass of scrambled configurations. This tendency, which reflects an asymmetry in the space of possibilities, is present whether changes take place by drift or by selection.

Yet, our claim to have identified an intrinsic tendency is unsatisfying, because we have not implicated a proper mechanism. Evolution does not look ahead: for every effect, there must be a blind, local cause. The doctrine of mechanism demands that we identify such causes. To invoke, as a causal mechanism, a global asymmetry in a possibility-space— a mere abstraction about future potentialities—would be to leave the mechanistic imperative unanswered.

The lack of a properly construed cause for intrinsic tendencies is an issue with implications beyond CNE. An argument about tendencies arising from abstract possibility-spaces has been made before, e.g., by Kauffman [27], or by McShea and Brandon [28]. Some evo-devo enthusiasts have declared a “third revolution” (after Darwin and the MS) [29] on the grounds that the potentialities of developmental systems represent local intrinsic biases that shape evolving organisms in ways not reflected in “reductionist” population genetics.

Such claims have been dismissed for precisely the reasons we would expect. In his stark rejection of evo-devo’s claim for novelty, Lynch writes "No principle of population genetics has been overturned by an observation in molecular, cellular, or developmental biology, nor has any novel mechanism of evolution been revealed by such fields" [10]. To satisfy Lynch, a “mechanism of evolution” must be expressed as a principle of population genetics, and thus developmental studies cannot reveal a “novel mechanism of evolution” (see also [30]). More generally, evolutionists have agreed for the past half-century that the locale of evolutionary causation is the population, and they have dismissed causal claims that were not rendered as population-genetic causes or forces.

The MS theory of causal forces [31] follows from a conception of evolution as “shifting gene frequencies” in the “gene pool”, a buffered system that maintains an abundant supply of infinitesimal allelic variation, so as to justify Darwin’s concept of natural selection (#1). In the MS, all of evolution, including macroevolution, can be reduced to shifting from one multi-locus distribution of allele frequencies to another. The fundamental forces of evolution— selection, drift, mutation and migration— are the processes that shift frequencies. A process is a “force” if it can change a frequency f to f + δ, where δ is an infinitesimal difference. Development is not a population process, thus it is not an evolutionary cause or force [30,32]. Importantly, all causes have the same kind of effect (a shift in frequency), and this common currency of causation makes it possible to combine and compare forces: selection is the strongest force, while mutation is the weakest; because mutation rates are so small, mutation cannot overcome the opposing force of selection; the force of drift becomes stronger in smaller populations.

Note that mutation occupies a dual place in the MS. Mutation may be conceived both as a mass-action pressure that shifts the frequencies of alleles already present, and as a process that introduces new alleles at random intervals. In the MS, the former concept fits the definition of a mechanistic cause or “force”, while the latter does not. This is why one may find, in a contemporary textbook, the otherwise nonsensical claim that “mutation's role as the source of genetic variation is usually more important than its role as a mechanism of evolution" [33], i.e., “mechanism of evolution” means “population-genetic force”. The architects of the MS stressed that mutation is the ultimate source of variation, but it is not a proximate cause of evolution (i.e., evolution does not begin with a new mutation), nor an effective agent of change: “mutation merely supplies the gene pool with genetic variation; it is selection that induces evolutionary change“ (p. 613 of [23]; for further documentation of this view, see [34]).

This view of evolutionary causes seems to offer little hope for understanding CNE. Based on the intuitive argument given above, we wish to identify an intrinsic source of directionality toward increased scrambling: but this cause is not the force of selection (because it is present when fixations happen by drift); nor of drift (because drift can’t favor one outcome over another); nor of migration (which isn’t involved); nor can it be the force of mutation (which is ineffectual if selection pressure is present).

Thus, either the intuitive argument we developed (above) to explain scrambling is wrong, or the theory of forces fails to describe a true cause that is right in front of our faces.

That cause is *bias in the introduction of variants*, and its lack of concordance with the classical forces theory arises because it is not based on the “shifting gene frequencies” view of the MS, which assumes abundant variation as a pre-condition for “evolution”, but on the mutationist conception of evolution as a 2-step proposal-and-acceptance process [35]. Such a 2-step process is subject to a “first come, first served” dynamic, such that a bias in the initial step (the introduction of a new allele by mutation) may impose a bias on outcomes [35].

**Figure 5:**
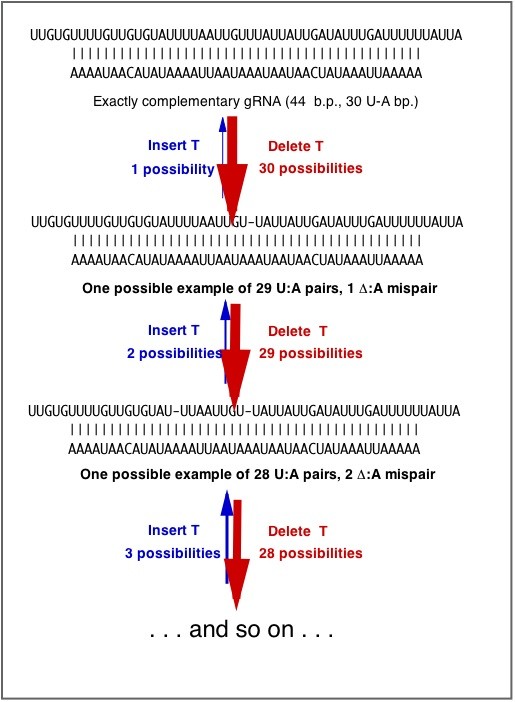
**Intrinsic biases that favor accumulation of editing sites. **In the CNE model (and, one might imagine, any model), new gRNAs (guide RNAs) arise fortuitously from anti-sense transcription of fragments of the parental gene. Initially, gRNAs are perfectly complementary to the mRNA of the parental gene: they play no role in editing and are subject to random loss. However, given a mechanism for editing the mRNA by inserting U’s complementary to A’s in the gRNA, the gene may evolve neutrally by events of mutation and random fixation that delete T’s, in which case the gRNA now becomes “functional”. A tendency toward accumulation of editing sites is expected due to biases in the introduction process. Initially, with 30 U:A pairs in the gRNA:mRNA region of complementarity, there are 30 ways to delete a T, each representing an evolutionary step toward editing. Once this happens, there is 1 way to revert, and 29 ways to delete a T, resulting in more extensive editing. Initially, increases in editing are favored, but over time, the system will equilibrate to an intermediate density of editing sites.

This fits our intuitive understanding of CNE. In the case of scrambled genes in Figure 4, a gene with 1 inversion has 1 mutational path to reduced scrambling, and 4 to increased scrambling. Thus, there is a 4-fold local bias in mutation that favors increased scrambling. In the CNE model for RNA pan-editing, incremental gain of editing sites is favored by the biases illustrated in Figure 5. Thus we have rendered a global bias in terms of a local population-genetic cause. Such a kinetic bias, of course, could be offset by a bias in the second step, which is fixation (or loss) by selection or drift, but this does not change the fact that it is a prior bias: it is not a side-effect of selective fixation, nor a limit on selective fixation, nor a constraint on selective fixation.

Mathematical formalisms of this 2-step view represent evolution in terms of events that follow origin-fixation dynamics, characterized by the product of (1) the rate at which some mutant type is introduced into a population and (2) the chance that a newly introduced mutant of that type will go to fixation. Such models have played an increasingly prominent role in evolutionary genetics over the past 40 years, with applications including Kimura’s Neutral Theory [36], the mutational landscape model of adaptation [37,38], Bulmer’s selection-mutation-drift model for the evolution of codon usage [39], much of Lynch’s thinking about genome evolution (e.g., box 6.2 of [40]), some detailed simulations of protein evolution (neutral [41] and adaptive [42]), the “mechanistic” models in the widely used PAML software for phylogenetic analysis [43], and so on.

Given a population of N individuals, a type of allele that emerges by mutation with a rate μ per gene per generation is introduced into the population at a rate 2Nμ (assuming diploids, i.e., 2 genes per individual), where it faces acceptance with probability π fixations per introduction. The bias in rates of two types of changes is then [34]

(1) $$\frac{R_{i}}{R_{j}}=\frac{2N\mu_{i}\pi_{i}}{2N\mu_{j}\pi_{j}}=\frac{\mu_{i}}{\mu_{j}}\times \frac{\pi_{i}}{\pi_{j}}$$

That is, the relative rate of the two types of changes jointly reflects one factor representing the relative rate of introduction μ_i_/μ_j_ (e.g., the bias in the first step of Figure 5 is μ_i_/μ_j_ = 30) and a second factor representing the relative probability of fixation, π_i_/π_j_. When i and j are classes of neutral variants in the strict sense, the probability of fixation is π_i_ = π_j_ = 1/(2N), and Eqn 1 reduces to μ_i_/μ_j_. For selective fixations, where a useful approximation is Haldane’s π_i_ ≈ 2s_i_, it follows that π_i_/π_j_ ≈ s_i_/s_j_. The verbal “other things being equal” assumption that we invoked earlier corresponds to the condition of s_i_ = s_j_, in which case, the overall bias reduces to μ_i_/μ_j_, just like the case for neutral evolution, even though fixations take place by selection!

For such a model to apply, the rate of introduction of new mutations (2Nμ) must be small, i.e., the “pressure” of mutation is low, which explains why they sometimes are called “weak mutation” models [44].

This 2-step view accounts for much of the novelty of the mutational landscape model underlying recent advances in the study of adaptation [15,37,44]. In this model, Eqn 1 is used as a “move rule” for deciding on the next adaptive step: i is a beneficial mutation of interest, j represents all beneficial alleles that can be reached in 1 mutational step, and the result is the chance of choosing i as the next step in an adaptive walk.

Importantly, Eqn 1 implies that the introduction process can bias the course of adaptation systematically. This particular implication is lost in Orr’s simplified version of the mutational landscape model, which assumes that mutation rates are all the same [15]. Thus, it is noteworthy that, in the experimental study of parallel adaptation by Rokyta, et al. [45] heralded as a critical test of the model (and indeed, as “the first empirical test of an evolutionary theory”) [38], Orr’s simplifying assumption is rejected, so as to allow transition-transversion bias in mutation, leading to a better fit with results. They also substitute an improved term for Haldane’s 2s in the probability of fixation. That is, the results of Rokyta, et al. justify the evolutionary relevance of both factors in Eqn 1, representing the two steps in a 2-step mutationist process.

## A step, not a shift

Some evolutionary thinkers (e.g., Gould [46]) take a reflexively Darwinian view of the evolution of evolutionary thought: the differences between theories (and the changes in mainstream thinking over time) are merely shifts in emphasis along pre-determined axes— a little more of this, and a little less of that (for an extreme example, see Figure 1 of [47]). According to this view, the advent of the Neutral Theory (for instance) signifies no conceptual novelty, but only a difference in the weight placed on “chance”.

Yet the actual history of evolutionary thought reveals the vital role of contested, conflicting, or otherwise non-shared concepts. As noted above, the MS endorses Darwin’s creative concept of selection (#1) with its “raw materials” view of variation, while rejecting the mutationist view (#3) with its emphasis on the distinctiveness of individual mutations. Thus Mayr, in a piece [48] entitled “The Resistance to Darwinism and the Misconceptions on which it was Based”, writes:

Those authors who thought that mutations alone supplied the variability on which selection can act, often called natural selection a chance theory. They said that evolution had to wait for the lucky accident of a favorable mutation before natural selection could become active. This is now known to be completely wrong. Recombination provides in every generation abundant variation on which the selection of the relatively better adapted members of a population can work (p. 38).

Given that “evolution” in the MS is defined as shifting from one multi-locus distribution of allele frequencies to another, the mutational introduction of new alleles is not part of “evolution”, except as an implicit pre-condition [35]. Accordingly, origin-fixation models played no role in the development and dissemination of the MS. Although they now represent an important (albeit unrecognized) branch of theoretical evolutionary genetics, they did not appear until the molecular revolution of the 1960s [49-51].

This has important implications for evolutionary reasoning. Evolutionists use the language of “forces”, believing that it provides a generalized framework for reasoning about evolution, even when origin-fixation dynamics are relevant. Sometimes the inappropriate use of the “forces” language is innocuous (e.g., [14]), but in other cases it leads to errors in reasoning.

In particular, the theory of forces leads to errors in reasoning about variation-biased evolution. The classical reasoning of Fisher and Haldane (for references and explanation, see [35]) rejects variation-induced trends (“orthogenesis”), on the grounds that, because mutation rates are so small, mutation pressure is a weak force easily overcome by selection. In effect, this argues that mutation may influence the course of evolution only by driving an allele to fixation against the opposing pressure of selection— which is roughly valid if one assumes that evolution is deterministic and that all of the alleles relevant to the outcome of evolution are present initially, i.e., if one accepts the MS.

Rather than rejecting this “opposing pressures” argument, contemporary evolutionists (as explained in [35]) have extended it to allow mutation-biased neutral evolution, on the grounds that mutation pressure can be effectual when the opposing pressure of selection is effectively absent (i.e., under neutrality). For instance, in the literature of molecular evolution, mutational explanations for patterns (e.g., GC content) are nearly universally described in terms of “mutation pressure”, are labeled “neutral”, and are considered rejected when there is evidence for selective allele fixations (e.g., [52,53]). Similarly, a seminal review on “developmental constraints” [54] suggests that developmental biases on the “production” of variation might bias evolution, but argues that this would be consistent with population genetics only if we allow the changes to be neutral, as otherwise they would be opposed by selection.

However, in the realm of origin-fixation dynamics, these are errors in reasoning: they result from applying a theory of causes that doesn’t fit, based on a theory of evolution that doesn’t fit. To suggest, by “opposing pressures” logic, that mutation-biased evolution must be neutral, is to imply fixation by mutation, as though mutation “pressure” drives alleles to fixation when selection is looking the other way. Yet fixation by mutation, given the low rate of mutation, is typically an absurdity that would take huge numbers of generations (e.g., this is ruled out explicitly in [55]). In neutral evolution, fixation takes place by drift, and the mutational bias is due to a bias in the introduction process, not to a bias in the fixation process. And mutation-biased evolution need not be neutral, as shown by Rokyta, et al.: the mutational bias on the outcome of parallel adaptation is clearly a bias in the introduction process, not the fixation process, and it cannot be explained by invoking the “force” of mutation, i.e., the mass-action pressure.

Evolutionists have been using mathematical equations of 2-step origin-fixation dynamics with increasing frequency for 40 years, while interpreting the results using an incommensurable “forces” theory from the MS, which deliberately rejected the 2-step view in the guise of mutationism [34]. The “forces” theory appears to allow variation-biased evolution under the condition of neutrality, but this appearance is superficial; and the “forces” theory can not explain (not even superficially) the case of mutation-biased adaptation shown theoretically in [35] and observed by Rokyta, et al. [45].

The theory of causes that we need (see also [34,56]) would tell us that evolution is a dual process of the introduction and sorting of variation in a hierarchy of reproducing entities, where reproductive sorting may be biased (selection) or unbiased (drift); non-randomness in the process may be introduced at either step, or both together; the common currency of causation is a bias in the odds that evolution will take one course versus another.

This view immediately suggests some different rules for reasoning, including statements about causation that *one will not find in any textbook*: mutation-biased evolution is not the result of the force of mutation pressure, but the result of a bias in the introduction process; it is not the largeness of mutation rates, but the smallness of the rate of introduction (2Nμ) that creates the potential for such biases to influence the course of evolution; this influence does not depend on neutrality, but can occur even when fixations take place by selection [35].

Importantly, the notion of a bias in the introduction process is broad. It does not necessarily reflect a global asymmetry in state-space, but may convey purely local effects of mutation, e.g., the role of transition-transversion bias in parallel adaptation cited earlier [45]; or of a bias in G→A vs. A→G mutation rates in emergence of certain drug-resistant HIV forms [57]. Also, it applies across any magnitude of bias, e.g., the dramatic reversals that we imagined previously are merely kinetically disfavored pathways, by virtue of relying on mutations with exceedingly low rates. In addition, it is not limited to effects of mutation *per se*, but may be applied to any feature, including phenotypes, thus developmentally mediated biases in variation [58] can be construed as true evolutionary causes of orientation [59]. Finally, one may conceive of an introduction process operating (with biases) at various levels of a hierarchy [56].

Two caveats are in order. First, one should not over-estimate the domain of origin-fixation dynamics, nor the relevance of mutationist thinking. While the notion of two perfectly distinct steps— origin and fixation— allows for conceptual clarity, and has encouraged useful models and software, it is an idealization, strictly applicable only in imaginary cases. Furthermore, the underlying conception is not a sufficient basis for a comprehensive theory. That is, the architects of the MS excluded mutationism and proclaimed that all of evolution could be reduced to “shifting gene frequencies”, but we must not make the complementary mistake of imagining that all of evolution can be reduced to an origin-fixation process or to the mutationist conception of selection (#3). While new mutations always are accepted or rejected, ultimately, they aren’t always accepted or rejected in such a way that evolution follows origin-fixation dynamics [60].

Second, to call into question the MS is not to call into question the body of purely analytical truths, known as theoretical population genetics, with which the MS is commonly confused. Instead, the arguments above relate to the MS as a conjectural theory, and to its verbal theory of causes. Relative to the purely abstract truths of theoretical evolutionary genetics, the essential claim of the MS (that genetics rationalizes Darwinism) is approximately equal to the conjecture that evolution can be understood as though its state-space were limited to the domain of classical quantitative genetics. This conjecture ultimately fails, yet there are some realistic cases in which selection drives quasi-continuous change in quantitative traits indefinitely, based on abundant small-effect variation from many loci, thus we need not discard Darwin’s concept of natural selection as a shape-shifting force. Likewise, we need not discard the concept of mutation as a mass-action force: there are realistic scenarios in which a mass-action pressure of mutation is the properly conceived cause of some effect, such as the loss of a gene [61], or the mutational cost of excess DNA in Lynch’s theory for increased genome sizes in species with small populations [40].

However, as a general theory of evolutionary causes, the “forces” view is insufficient, and this insufficiency is not remedied by adding chance, contingency and constraints, which are vague explanatory principles, not causes. “Chance” obviously is not a force or cause. “Constraint” is not a force, nor is it any positive cause, but a condition indicating that an imaginary ideal is unsatisfied. “Contingency” is likewise not a cause, but a conceptual placeholder indicating the inapplicability of an ahistorical idealization in which systems reach global equilibria independent of their initial conditions. Patching up the MS with constraints, chance and contingency expands its scope to cover a wide range of cases outside of the core paradigm, yet this expansion entails such an enormous loss of rigor and clarity that the result does not deserve the name of “theory”. Is there anything— evolution, politics, planetary motions, bridge-building— that cannot be explained by the theory of population-genetic forces when it is combined with the 3 catch-all principles that outcomes are contingent on initial conditions, constrained by various factors, and subject to chance?

## Conclusion

The concept of constructive neutral evolution (CNE) was proposed originally to have both a direct significance (as a schema for generating neutral models for complexity) and an indirect significance, as a conceptual tool for exploring the evolutionary role of factors other than positive selection. In the former guise, CNE models provide an alternative to adaptive rationales for some cases of seemingly inordinate complexity. This aspect of CNE will be of interest to those working on systems such as RNA pan-editing or gene-scrambling.

For the rest of us, CNE exposes an alternative conception of evolutionary causes. This conception is not based on the “shifting gene frequencies” paradigm of the MS, but instead partakes of the mutationist view of evolution as a 2-step proposal-and-acceptance process whose kinetics depend directly on the introduction of novelty by mutation-and-altered-development. This conception, which is not unique to CNE, now lays claim to important theoretical, experimental, and practical results bearing on the kinetic link between evolution and mutation. Its most striking result is the potential for mutation-biased adaptation shown theoretically in [35,42] and observed in [45]. Its most provocative implication is that developmentally mediated biases in the introduction of phenotypic variants represent a legitimate evolutionary mechanism that the MS fails to recognize, not because the MS is reductionistic, but because its essential commitment to rationalizing Darwin’s creative shape-shifting concept of natural selection leads to a restrictive view of evolutionary causation in terms of mass-action forces or pressures.

This innovation has emerged without fanfare, its significance masked by its historical association with molecular sequences and with neutrality.

Nevertheless, it would be a mistake to conclude that is has no particular significance. The evolutionary 2-step shares many of the implications of the “mutationist” view that appealed to the founders of genetics [34], but was excluded from the 20^th^ century neo-Darwinian consensus due to its lack of concordance with Darwin’s views. The result is a curious disconnect between what some of our most interesting and productive models imply about evolution, and what our textbooks say. The MS theory of causal forces urges us to choose between the opposing pressures of mutation and selection. By contrast, within the 2-step view, we can understand evolution differently, as both an expression of internal tendencies of variation and, at the same time, a response to conditions mediated by differential reproduction.

## Competing interests

The author declares that he has no competing interests.

## Reviewers’ comments

The author notes that, in response to comments from my colleague David McCandlish, I have made a minor change of wording in one sentence, and I have deleted a rhetorical question that had a misleading implication (regarding the reasons that Orr quotes Bateson’s statement on the sizes of changes in evolution).

Reviewer’s report 1

Alex Kondrashov, University of Michigan

It is hard for me to provide a useful review for this manuscript, because it consists, almost exclusively, of rather general reasoning, instead of precise, falsifiable statements. I do not see much value in almost-philosophical consideration of “Modern Synthesis” and “neo-Darwinism”, whatever these expressions might mean. I fully agree with the original claim of the author, made in 1999, that fixations of effectively neutral alleles may result in something new and that limitations imposed by availability of mutations on the outcomes of adaptive evolution are crucial (if becoming humans depended on inserting a specific segment of 10 amino acids into a particular site of a particular protein, we would remain apes forever, because the probability of such an insertion is vanishingly low). Statements of this kind were made many times (e.g., [62]), but here I am not concerned about priority. Instead, I do think that our understanding of evolution at the level of sequences already matured past the phase when discussions of basic principles are useful for professionals. We all believe in mutation, selection, and drift, I hope.

***Author’s response:****Kondrashov’s position is that professional scientists already agree on the implications of selection, mutation and drift, and have no need of the kind of “discussions of basic principles” found in my article. In other words, to the extent that my article explains implications of theoretical evolutionary genetics, and interprets results in terms of abstract causal processes, Kondrashov does not object, but finds it trivial, on the grounds that everyone knows this already. Where others might see complex schemes, difficult concepts, and problematic choices, Kondrashov sees the same basic principles underlying everything. Thus, he sees no novelty in my 1999 paper, but only the same principles underlying many prior sources.*

*The aspiration to derive a common understanding of causation from basic, universally accepted principles may be a laudable ideal, but as a description of reality, this view is indefensible. The reality of scientific practice is that selection, mutation and drift are not basic principles with universally agreed implications, but complex concepts freighted with history and metaphor, whose implications are disputed and problematic. For instance, if one simply consults the contemporary Oxford Encyclopedia of Evolution, the topic article*[63]*provides multiple conflicting descriptions of natural selection, including as a “theory” entangled with Darwinian assumptions (*e.g.*, “natural selection differs from most alternative theories of evolution in the independence between the processes that direct variation and that direct evolution”; “In evolution by natural selection, the processes generating variation are not the same as the process that directs evolutionary change. Variation is undirected”). Evidence that the implications of such concepts are not a matter of universal agreement is given in my article,* e.g.*, cases in which evolutionary biologists make mistakes due to the influence of an “opposing pressures” argument that goes back to Fisher and Haldane. I listed several principles of causation that follow under origin-fixation conditions, and stated that these principles will not be found in any textbook (which is perhaps my article’s only “precise falsifiable statement”, albeit a meta-scientific one).*

Thus, while Kondrashov may find my comments about evolutionary causes rather obvious, I doubt that they will be so obvious to everyone, and clearly they have not been obvious in the recent history of our field.

Reviewer’s report 2

Eugene Koonin, National Center for Biotechnology Information

In my opinion, this is a truly excellent article that presents an exceptionally clear and well-written account of constructive neutral evolution, its underlying mechanisms and implications. A fully convincing case is made for the major role of CNE in evolution and ‘creative’ role of mutational biases. I particularly like the simple entropic argument for the virtual inevitability of the emergence of ‘complexity’ (or simply mess) via CNE. I have no objection to any of the statements in this article. I would only like to add that the contribution of mutations to evolution does not stop at biases. It is clear now that a variety of mechanisms exist for directing mutations to specific targets that are relevant for adaptation under the given conditions. This results in a plethora of Lamarckian and pseudo-Lamarckian processes that substantially contribute to evolution [64].

***Author’s response:****As Koonin helpfully notes, the concept of "mutation bias" (which merely implicates non-uniformity) is distinct from the idea of non-uniformities in variation that arise from programmed responses to conditions, and thus evoke the ideas of Lamarck.*

While my article may have convinced some of the “major role of CNE in evolution”, I was aiming only to explain it more fully. CNE is an abstract schema for generating neutral models, and a conceptual tool for exploring ideas about causation and explanation. In the former guise, it suggests some interesting models that are largely ignored, except in the case of gene duplication (due to the independent efforts of Lynch and colleagues). The adaptationist program tends to ensure that, to the extent that neutral models are apt, their distinctive conditions and dynamics are incorporated into the next generation of adaptive models. My hope is not that CNE will win a greater share of the complexity market, but that it will cause us to re-think how we frame evolutionary questions (and answers).

Reviewer’s report 3

Johann Peter Gogarten, University of Connecticut

I first read about Constructive Neutral Evolution (CNE) in Arlin Stoltzfus's 1999 paper "On the possibility of constructive neutral evolution" [7] – I had missed Covello and Gray's earlier description of this concept [6]. Having been nurtured on a steady diet of adaptive reasoning during my education, I found Arlin Stoltzfus's description refreshing and convincing, and I have been a fan of neutral pathways towards complexity ever since. Other earlier writings by Arlin Stoltzfus that I highly enjoyed reading were the guest blogs he wrote for Larry Moran's Sandwalk blog [65], analyzing the forces that shaped today's perception of the modern synthesis and neo-Darwinism.

Both of these topics, CNE and the conflict between proponents of the modern synthesis and the mutationists, are reviewed in the current manuscript. The manuscript then discusses the concept of forces acting on gene frequencies and provides a clear discussion of bias in the introduction of variants, which is better understood as a two step proposal-and-acceptance process. I particularly liked the demonstration that mutation biased evolution need not be neutral evolution. My only suggestion for improvement concerns the initial description of selection in the mutationist framework as "a stochastic filter (a sieve or pruning hook) that modulates the chance of survival of spontaneously arising mutants, some of which get lucky". A little bit more details and explanation will make it easier for the reader to parse this rather dense description.

And in equation 1 the μ_i_ in the denominator on the right hand side of the equation should be μ_j_.

***Author’s response:****I have made the suggested correction in Eqn 1. In regard to understanding the mutationist concept of selection as a sieve, it may be helpful to note that this was offered (historically) to contrast the Darwinian notion that variation supplies only raw materials for a creative force of selection that shapes a mass of variations, the way that a human hand would be said to shape a sand-sculpture— the character of individual grains of sand is unimportant in this process, both because the grains are small relative to the whole, and because they are never moved individually. The key aspects of the mutationist conception are that (1) the issue is framed in terms of the ultimate fate of distinctive individual mutants, (2) the role of “selection” is to modulate this fate, and (3) this modulation is stochastic,* i.e.*, beneficial variants are not guaranteed of success, nor are unfavorable (or neutral) ones guaranteed of failure. Statements from Morgan and others that document this view may be found in*[34].

## Supplementary Material

Additional file 1 This Perl script will compute a graph showing the inversion paths between all (scrambled and unscrambled) configurations of a segmented gene (for help, type " ./scramble_space.pl – help"). Click here for file
